# Developing Traits of Self-Confidence and Intrinsic Motivation in Students with Severe Special Educational Needs in Physical Education Lessons

**DOI:** 10.3390/bs15111449

**Published:** 2025-10-24

**Authors:** Simas Garbenis, Irena Kaffemaniene

**Affiliations:** Institute of Education, Šiauliai Academy, Vilnius University, LT-76352 Šiauliai, Lithuania; irena.kaffemaniene@sa.vu.lt

**Keywords:** self-confidence, intrinsic motivation, emotional intelligence, micro-ethnography, students with severe special educational needs

## Abstract

This study, by analyzing processes taking place in physical education (PE) lessons, sought to identify how traits of self-confidence and intrinsic motivation developed in pupils with special educational needs (SEN). The research employed a micro-ethnographic study that was directed at the research object: opportunities to develop pupils’ traits of self-confidence and intrinsic motivation. Empirical data were gathered through video recordings of PE lessons. The sample comprised 36 first-grade pupils with severe SEN. Using reflexive thematic analysis, we identified themes and subthemes that revealed the development of emotional intelligence traits: self-confidence and intrinsic motivation, as well as themes and subthemes that revealed the manifestation of these traits in PE lessons. The study found that constructive dialogic interaction—emphasizing emotional support and strength-oriented, reciprocal teacher–pupil reflection—was the key factor in developing both self-confidence and intrinsic motivation. The study revealed distinctive features of self-confidence (demonstration of self-efficacy, positive self-assessment, reflection on the perception of strengths) and intrinsic motivation (choosing challenging tasks, determination and persistence, the need to improve skills and achieve better results). The empirical findings reflected universal methods for cultivating emotional intelligence traits that could be transferred to other educational contexts. The article presents a small part of the dissertation research data.

## 1. Introduction

**Trait emotional intelligence.** The construct of trait emotional intelligence describes a person’s perceptions of their individual traits and dispositions—emotional resilience, adaptability, self-efficacy, and other emotion-related aspects (Petrides & Furnham, 2001; Petrides et al., 2016; Petrides & Mavroveli, 2018). Alternatively, the construct is described as trait emotional self-efficacy (Petrides et al., 2016; Petrides & Mavroveli, 2018), indicating links with self-efficacy theory (Bandura, 1997). It is emphasized that trait emotional intelligence reflects an individual’s subjectively perceived abilities but does not necessarily represent actual characteristics (Petrides & Furnham, 2001). By contrast, the alternative ability model conceptualizes emotional intelligence as a set of cognitive abilities (Mayer et al., 2016).

Trait emotional intelligence theory is one of the principal approaches to emotional intelligence in contemporary scholarly literature and is widely used in empirical research. It has been applied across clinical, organizational, and educational domains (Petrides & Mavroveli, 2018). The theory is frequently employed in studies with children and adolescents because, by definition, the structure of emotional intelligence differs across pupils, adolescents, and adults (Andrei et al., 2015; Güler & Turan, 2022; Özal et al., 2024). Considering the sample of this research, it is important to note that, for pupils, the structure of emotional intelligence comprises nine traits: adaptability, affective disposition, emotion expression, emotion perception, emotion regulation, low impulsivity, peer relations, self-esteem, and self-motivation (Petrides & Mavroveli, 2018). A clearly delineated trait structure for children versus older populations provides a strong rationale for conducting similar studies with younger pupils, as it enhances the accuracy and reliability of the findings.

Trait emotional intelligence theory has also been used—albeit infrequently—in samples comprising SEN pupils. For example, Mavroveli and Sánchez-Ruiz (2011) reported that SEN pupils scored lower on trait emotional intelligence than their neurotypical peers. Similarly, Abo Elella et al. (2017) found that pupils with ADHD demonstrated lower trait emotional intelligence scores.

Taken together, these studies suggest that trait emotional intelligence theory captures characteristics that are required for pupils’ everyday functioning on an ongoing basis, rather than framing emotional intelligence solely as traits that must first be identified and then applied selectively.

**Conceptualization of self-confidence and intrinsic-motivation traits:** In this study, drawing on trait emotional intelligence theory, self-confidence (self-esteem) is conceptualized as an individual’s positive self-assessment and belief in their ability to overcome challenges and achieve success (Bhat & Arumugam, 2021; Manikandan, 2015), while intrinsic motivation (self-motivation) is understood as an individual’s interest in, engagement with, and satisfaction derived from an activity (Ryan & Deci, 2020; Bandhu et al., 2024). A more detailed explication of these concepts is provided below.

In trait emotional intelligence theory, self-confidence is variously defined, for example, as self-esteem—one’s perception of personal worth (Petrides & Furnham, 2001; Petrides et al., 2016; Petrides & Mavroveli, 2018). Other authors similarly define self-confidence: belief in one’s personal value and likelihood of success (Manikandan, 2015); belief in one’s ability to overcome challenges and achieve success, and the willingness to act accordingly; a positive yet realistic view of one’s abilities (Bhat & Arumugam, 2021); confidence in one’s competence in a specific field, context, or situation (Maclellan, 2014); and opinion of one’s own academic and social abilities and ability to attain goals (Upadhyay et al., 2020).

Self-esteem is a component of emotional intelligence (Petrides et al., 2016) and a part of self-concept—the degree to which one positively evaluates one’s own traits and characteristics (APA, 2023). It reflects how a person evaluates themselves with regard to their own and others’ expectations; it is a mental disposition that prepares individuals to act according to their expectations of success (Vărăşteanu & Iftime, 2013; Sharma & Sharma, 2025). Closely related to self-esteem is belief in success, grounded in prior experience of achieving goals and personal outcomes; a positive attitude toward oneself “attracts” success and thereby reinforces self-confidence (Vărăşteanu & Iftime, 2013).

Perkins (2018) proposed an integrated model of self-confidence as a multifaceted construct, distinguishing internal and external self-confidence. The author argues that internal self-confidence is influenced by three factors: self-efficacy, self-esteem, and self-compassion, which protects against negative emotional consequences associated with perceived failure (APA, 2018). Thus, self-confidence encompasses emotional, behavioral, and cognitive components (Perkins, 2018). According to Perkins (2018), the most noticeable and identifiable signs of self-confidence include emotionality/optimism, willingness to take risks and initiative, nonverbal and verbal communication, independence of thought and action, and trust in one’s own judgments and evaluations.

Some studies show that self-efficacy correlates with self-confidence (Blanco et al., 2020; Malureanu et al., 2021; Inagaki, 2022), among others. It has also been established that self-confidence is positively and significantly correlated with both intrinsic and external motivation. Intrinsic motivation is characterized by interest and satisfaction with the activity (Ryan & Deci, 2020; Bandhu et al., 2024). Although, depending on the context and individual, external rewards may also influence motivation (Ryan & Deci, 2020), intrinsic motivation nevertheless promotes greater engagement, creativity, and well-being (Bandhu et al., 2024).

A concept related to, but not identical to, self-confidence is self-efficacy, which refers to an individual’s belief that they can perform tasks in a given domain to a certain level (Bandura, 1997; Bandura, 2012). People’s beliefs in their capabilities differ across domains, situations, and contexts. Self-efficacy is considered a situation-specific expression of self-confidence (Maclellan, 2014).

The perception of self-efficacy—one’s ability to learn or to act—is a cognitive process in which individuals use various sources of information (personal mastery experiences, social persuasion, etc.) to evaluate or confirm their self-efficacy (Schunk & DiBenedetto, 2020). Beliefs about one’s efficacy influence motivation to overcome difficulties and achieve goals (Bandura, 2012; Bandhu et al., 2024; M. Jansen et al., 2015). From the perspective of social-cognitive motivation theory, motivation is affected not only by external stimuli but also by internal cognitive factors—beliefs, expectations, self-perception, and self-efficacy; thus, self-efficacy is one of the essential aspects of motivation (Bandhu et al., 2024).

**Research on pupils’ self-confidence and motivation**: Research shows that self-confidence is associated with success, learning outcomes, competencies, and personal well-being (Perkins, 2018). Thomaes et al. (2017) found that most children evaluate themselves very positively. As cognitive abilities mature, children begin to understand that their self-view is not always realistic; nonetheless, their self-concept remains positive due to social norms favoring positive self-views and because of a self-protection motive to avoid self-negativity (Thomaes et al., 2017).

Self-confidence is a key to life success, including for children with special needs (Setiawan et al., 2023). Insufficient self-confidence may indicate that learners doubt their internal resources (abilities, endurance, ingenuity) to complete a task successfully. Conversely, excessive self-confidence may reduce motivation to learn and improve (Maclellan, 2014).

A study of emotional intelligence and academic motivation among primary-school pupils revealed a positive and significant correlation between the two variables (Arias et al., 2022). Similar results were found in research on the relationship between emotional intelligence, emotional well-being, motivation, and learning strategies (Nieto-Carracedo et al., 2024).

Studies have established that the structure of emotional intelligence in students with special educational needs does not differ from their peers without such needs. However, students with special educational needs display lower emotional intelligence scores, which correlate with their academic attainment and behavioral characteristics (Mavroveli & Sánchez-Ruiz, 2011), with negative consequences at both personal and interpersonal levels. It is therefore plausible that some pupils with SEN may find it more difficult to deploy emotional intelligence abilities effectively, even if they possess them. Students with special educational needs more often feel lonely and rejected (Gaspar et al., 2016; Goldan et al., 2022) and have lower levels of self-confidence than their peers (Gaspar et al., 2016; Koukou et al., 2018). It has been shown that students with special educational needs have less confidence in their academic abilities (Hall & Webster, 2008, cited in Kim & Kutscher, 2021).

DeVries et al. (2022) found that perceived inclusion and academic self-concept differ by gender, perceived difficulties, and special educational needs. For example, studies by Maïano et al. (2019) and Douma et al. (2025) found a substantially lower level of global self-concept in students with intellectual disabilities compared to those without such disabilities. Children with special educational needs felt less included and reported lower academic self-concept (DeVries et al., 2022). The study by Nousia et al. (2022) showed that children with language disorders experience low self-esteem and perceive their school behavior as inappropriate only when their perceived academic competence is low. Perceived academic competence was associated with children’s stuttering and their self-esteem and perceptions of their behavior at school.

A scoping review by Wiliyanto et al. (2025) shows that students with learning disabilities experience negative self-evaluation, which may affect their motivation and self-confidence. Research has established that self-compassion has a significant effect on students with disabilities, contributing to better academic achievement, lower self-criticism, reduced stress, depression, anxiety, and loneliness, greater self-confidence, more favorable learning motivation, and better social well-being. These findings underscore the strong influence of self-compassion on the learning and social functioning of students with disabilities: with high levels of self-compassion, students can improve their academic results and social well-being (Wiliyanto et al., 2025).

A positive relationship has been identified between the level of self-confidence, learning motivation, and achievements in physical education among primary-school students (Mulya & Lengkana, 2020). There is evidence that motivation significantly affects the self-confidence of children with special educational needs (Setiawan et al., 2023).

**Fostering traits of self-confidence and motivation**: From a social-constructivist perspective, students construct their knowledge through active participation in their own learning, where the teacher acts as a mediator who creates and sustains a learning and problem-solving environment based on dialog and collaboration, and provides consultation, encouragement, and support to develop and evaluate learning (Bada & Olusegun, 2015).

Studies indicate that learners’ self-confidence can be developed (Christiansen et al., 2018; Maclellan, 2014; etc.). Maclellan (2014) argues that self-confidence is fostered by teachers’ attention to learners’ engagement in socially planned learning activities that encourage non-academic self-concept; by teachers’ dialogic feedback with students so that they reflect on non-academic self-concept, self-efficacy, and self-regulation; and by practicing metacognitive activity in every lesson.

There is a paucity of research on developing self-confidence and motivation as traits of emotional intelligence in students with special educational needs. A few earlier studies examined levels of emotional intelligence and interactions among its traits. However, there is a lack of scientific information about factors related to the development of self-confidence and intrinsic motivation among students with special educational needs (Kim & Kutscher, 2021).

In this study, trait emotional intelligence theory was adopted for several reasons. First, the studies reviewed above clearly highlight the relevance of—and the problem space for—developing self-confidence and intrinsic-motivation traits in pupils with special educational needs (SEN). These traits are distinguished within the trait emotional intelligence structure for children aged 7–12, and that structure is widely applied in educational research (Özal et al., 2024). It is also important to emphasize that, because these traits form part of a broader structure, they are likely to resonate with its other components; consequently, fostering one set of traits may yield effects that extend to other traits within the same structure.

**Characteristics of the physical abilities of pupils with special educational needs and the practical significance of physical education.** Studies by various authors indicate that children with special educational needs (owing to intellectual disabilities, autism spectrum conditions and other disorders) experience not only cognitive difficulties but also delayed motor development and lower levels of motor skills than their typically developing peers (Căpriţă & Balinta, 2023; Lloyd, 2016; Rintala & Loovis, 2013; Maïano et al., 2019; Müller et al., 2024). They may face challenges in developing both gross and fine motor skills, hand–eye coordination, balance, posture and strength. Motor difficulties can manifest during activities such as running and jumping.

Research has shown that children with motor impairments exhibit more emotional and behavioral problems (Rodriguez et al., 2019; Leisterer & Jekauc, 2019). A review of the scholarly literature reveals that pupils with special educational needs experience learning failures and psychological difficulties, and tend to have lower self-confidence, poorer quality of social relationships, and weaker adaptive abilities (Fink et al., 2015; D’Amico & Guastaferro, 2017; Lereya et al., 2023; Marzec, 2017; Tenerife et al., 2022; Smogorzewska et al., 2018). Their emotional intelligence indicators have been found to be lower (Mavroveli & Sánchez-Ruiz, 2011; Munday & Horton, 2021; Duchyminska et al., 2022), particularly in the areas of emotional expression, emotion regulation, socialization, impulse control, and adaptive behavior (Sandu & Bîrzu, 2023). Self-confidence is especially important in academic settings because it helps pupils realize their potential, feel capable, and remain motivated to engage willingly in activities (Koukou et al., 2018). For pupils with special educational needs, low self-confidence may arise not only from difficulties completing certain learning tasks or taking part in various activities, but also from lower levels of emotional intelligence. Physical education provision can improve pupils’ motor, cognitive and social skills (Suwiwa et al., 2025; Christiansen et al., 2018), among other benefits.

Most of these difficulties can be reduced or prevented through targeted emotional intelligence development, which can be implemented via physical activities. A systematic review by Rico-González (2023) showed that emotional intelligence can be developed in physical education (PE) lessons using purposefully designed programs. Other researchers (Fernandes et al., 2024) report that participation in sporting activities—even without explicit EI-focused programs—improves emotional intelligence indicators associated with life satisfaction. More frequent engagement in physical activity helps primary-school pupils develop their social–emotional competences (Wang et al., 2025).

In this study, by analyzing processes occurring in physical education lessons, we sought to identify factors that may influence the development of students’ self-confidence and intrinsic motivation. The manifestation of these traits was analyzed based on observable emotional and behavioral responses across different lesson activities. The study did not aim to measure changes in these emotional intelligence traits.

**The purpose of this study** was to analyze the factors and manifestations of the development of self-confidence and intrinsic motivation traits in students with severe special educational needs during physical education lessons.

**Research questions (RQs):**

RQ1: In which physical education lesson activities do students develop traits of self-confidence and intrinsic motivation?

RQ2: Which actions by the teacher help students develop traits of self-confidence and intrinsic motivation?

RQ3: How are students’ traits of self-confidence and intrinsic motivation manifested in physical education lessons?

## 2. Materials and Methods

**Theoretical foundation of this research**: The study is grounded in trait emotional intelligence and social-cognitive motivation theories. The processes of developing self-confidence and intrinsic motivation are considered from a social-constructivist perspective.

Social-cognitive theory integrates cognitive, behavioral, and social aspects of motivation. It emphasizes beliefs about self-efficacy—one’s belief in one’s abilities—which helps explain motivation by recognizing the influence of perceived competence on motivation to engage in particular activities (Bandhu et al., 2024).

Drawing on social constructivism—particularly claims about the construction of knowledge and identity through social interaction and cultural contexts (Berger & Luckmann, 1991)—we examined how, through social interactions in physical education lessons, the emotional intelligence traits of self-confidence and intrinsic motivation are developed.

**Research context**. The study was conducted in a special school in Lithuania. Data were collected in physical education lessons, without changing the content or structure of the lessons. Data were gathered in the natural physical education environment, adhering to the goals of physical education set out in the official Lithuanian general education curriculum, ensuring continuity in content (curriculum and lessons) and in teaching methods adapted and individualized according to students’ special educational needs. Lesson content comprised warm-up tasks, learning, improvement (refinement), and conditioning (development) tasks, and self-assessment activities.

**Methodology**. We adopted a micro-ethnographic methodology. Micro-ethnography is a qualitative method that uses video recordings as a key data source; the researcher focuses on moment-by-moment interactions, actions, behaviors, or communication to answer the research questions (Bayeck, 2023).

In this study we analyzed processes of developing self-confidence and intrinsic motivation among primary-school students with special educational needs in physical education lessons by examining activities, teacher–student interactions, and manifestations of students’ self-confidence and intrinsic motivation within those activities and interactions. The micro-ethnographic technique enables multiple viewings and analyses of captured moments of social interaction and students’ emotional reactions and supports targeted interpretation.

Micro-ethnography requires the researcher’s continued presence in the field (Bayeck, 2023). The researcher partially becomes a participant: being constantly present and collecting data, they participate in, observe, and reflect on ongoing processes (Garcez, 2017; Herrle, 2020). As noted, one of the authors is a physical education teacher who knows the students and the educational context exceptionally well; this enabled an in-depth exploration of the phenomenon—developing emotional intelligence traits in the natural lesson environment. Micro-ethnography is well-suited to school lesson contexts, which are clearly structured, have a particular microclimate, and involve small student groups with continuous social interactions.

**A section from the researcher’s self-reflexivity** is provided to both support the importance of the teacher–researcher role during this research and the research context as well: *Having worked in the field of physical education for a long time, I found it straightforward to implement the physical education curriculum. However, in teaching students with severe special educational needs I encountered difficulties related to the students’ emotional sensitivity and emotion regulation while performing various tasks. This challenge prompted interest in these students’ emotional self-awareness, emotional abilities, and difficulties. With a professional responsibility and close relationship with my learners—who call me by my first name—I felt compelled to help them not only achieve curriculum outcomes, but also to overcome emotional difficulties that I observed beyond lessons as well. The question arose: how can we foster traits of emotional intelligence in primary-school students with severe special educational needs during physical education lessons? I began studying the literature on emotional intelligence, expanding my understanding of its concepts, theories, and educational perspectives specifically for such students. This ultimately led me to doctoral studies to investigate, empirically, the process of developing these students’ emotional intelligence traits.*

**Empirical data collection methods**. To observe and record processes occurring in physical education lessons, we used video recording (Huber, 2020). Recordings were made in classes of 7–8-year-old students using two cameras: one capturing the whole class, and another mounted on the researcher’s chest to obtain higher-quality footage of individual interactions with students and peer interactions. Recordings were made from November 2020 to June 2021. In total, 288 physical education lesson recordings (11,525 min) were made; each recording lasted about 40 min and included all lesson parts (introductory, main, and concluding).

**Data analysis methods**. Empirical data were analyzed using reflexive thematic analysis (RTA) (Braun & Clarke, 2021; Braun et al., 2023) of the video recordings (Huber, 2020).

RTA is an interpretive method that identifies themes or other structures in qualitative data (Braun & Clarke, 2021; Braun et al., 2023), emphasizing the researcher’s active role in knowledge construction (Braun & Clarke, 2019; Braun & Clarke, 2021; Clarke & Braun, 2017). We combined inductive (“data-driven”) and deductive (“theory-driven”) RTA (Braun et al., 2023; Byrne, 2022; Braun & Clarke, 2019) and open coding grounded in visible participant actions and behaviors with latent coding grounded in inferred attitudes and feelings (Braun et al., 2023).

During initial viewing, we selected episodes that likely reflected processes of developing the emotional intelligence traits of self-confidence and intrinsic motivation (students’ emotional expressions, behaviors, teacher–student interactions). Episode analysis (sequential analysis) sought to identify manifest and latent meanings in audiovisual data (Huber, 2020), recurring interactions, communicative features, and challenges (Herrle, 2020), and to interpret the meanings of participants’ actions.

**Criteria for selecting video episodes for analysis:**

Self-confidence trait episodes—inclusion/exclusion criteria:

(1) Episodes capturing teacher–student social interaction during lesson activities (e.g., the teacher, considering students’ abilities, provides individualized support, encourages task completion, and guides (self-)assessment of progress or results). Episodes without active social interaction were excluded.

(2) Episodes where the teacher fosters students’ positive self-perceptions by providing feedback on demonstrated progress, achieved results, or appropriate engagement and participation; by encouraging and praising; or by helping students understand their strengths. Episodes were excluded if students did not demonstrate back-channels indicating emotional reaction, understanding of feedback, self-confidence, or awareness of strengths.

(3) Episodes where, during peer interactions, students (self-)assess their own and/or peers’ progress or results. Episodes were excluded if peer interactions only triggered emotional expression without (self-)assessment of progress or achievement.

Intrinsic motivation trait episodes—Inclusion/exclusion criteria:

(1) Episodes capturing students’ self-initiated desire to perform tasks, overcome difficulties, and try new possibilities. Episodes were excluded if students showed no desire to perform tasks, chose only acceptable/familiar tasks at usual requirements, or sought only external reinforcement.

(2) Episodes where students readily used opportunities to choose between more difficult and easier tasks and chose more difficult ones, or where they themselves introduced additional elements of difficulty, actively engaged, and strove to overcome challenges. Episodes were excluded if students desired tasks only when acceptable to them or avoided tasks due to perceived inappropriateness or intensity.

Using these criteria, we assigned preliminary codes to episodes, from which we later generated initial themes and subthemes and created thematic maps showing interrelationships.

The principal researcher independently reviewed the video recordings and transcribed them. The second researcher then systematically and repeatedly read the transcripts of episodes selected according to jointly agreed criteria and conducted the coding. The coding was subsequently discussed by both researchers and reconciled.

**A note from the researcher’s reflexivity:** *Having segmented the required data into episodes, I chose to analyze their content using an approach that treats the teacher–researcher’s subjectivity as a strength. In interpreting the episodes, I employed reflexive thematic analysis (RTA), which enables the organization of data into sub-themes and themes, while acknowledging the teacher–researcher’s subjective perspective as a natural, value-adding element of the analytic process.*

**Conducting RTA**: Following a reflexive approach, themes were not pre-defined; rather, they were generated from empirical data by grouping meaningfully related units (Braun & Clarke, 2019). We applied the six-phase RTA process (Braun & Clarke, 2006; Braun et al., 2023):

• Familiarization: initial and repeated viewing; transcription; note-taking on salient processes; selection of episodes for further analysis;

• Coding: open coding of transcript segments based on recorded meanings (interaction, emotion, etc.) aligned with indicators of self-confidence and motivation;

• Initial theme generation: exploring relationships among codes and developing a preliminary thematic map;

• Review and development of themes: refining relationships between themes and codes, considering didactic characteristics of tasks, program components, lesson goals, and sample specificities;

• Theme definition and naming: elaborating conceptual content and naming subthemes;

• Reporting: systematically describing analysis results, linking themes/subthemes with transcript examples and methodological reflections, and interpreting findings in light of existing research.

Reflexivity is a vital aspect of qualitative research (Braun & Clarke, 2021; Braun et al., 2023). In this study, reflections on the primary author’s teaching and research experience were important. The second author, experienced in qualitative methods, also provided reflections related to interpreting the thematic analysis results.

**Research sample**. In this micro-ethnographic study, a convenience sample was used for data collection, determined primarily by accessibility: the participants were the teacher–researcher’s pupils, selected with reference to purposive, criterion-based sampling. Accordingly, the analytical results pertain only to the selected sample of pupils with special educational needs (SEN). Inclusion criteria: students who (1) were in Grade 1, aged 7–8; (2) had special educational needs; and (3) had attended the study institution for at least one year.

Applying these criteria, we formed a sample of 36 first-grade students with special educational needs: speech and language disorders (19), attention disorders (5), complex disorders (4), learning disorders (2), attention and hyperactivity disorders (2), and emotional disorders (1 student). Analyses focused on the shared feature (severe special educational needs), without stratifying by specific diagnoses.

**A note from the researcher’s reflexivity:** *The scientific literature reviewed foregrounds a key point: irrespective of a pupil’s specific diagnosis, their emotional self-perception and emotion-related needs tend to be more acute than those of pupils without difficulties. I observed the same when comparing my practice in the private sports sector with my work in school. When presenting the research problem and core ideas to colleagues and advisers, some noted that a sample comprising pupils with severe special educational needs might be overly broad, difficult to manage or to delineate. However, both my professional experience and the literature indicate that pupils exhibit largely similar emotion-related difficulties, regardless of how they are categorized or characterized. For this reason, the micro-ethnographic data were not analyzed according to pupils’ specific diagnoses/conditions.*

**Research ethics**. The study adhered to general research ethics principles: reliability in ensuring quality (design, methodology, analysis, use of resources), honesty, respect for participants, and accountability (ALLEA, 2023). Informed consent, confidentiality, and anonymity were upheld (BERA, 2018), along with special ethical principles concerning children’s participation (Graham et al., 2015).

Given participation by primary-school students with special educational needs, additional principles relevant to sensitive social groups (children with developmental disorders) were applied (Jenkin et al., 2015). Parental/guardian consent was mandatory. Prior to the study, the researcher considered that, should parents or students decline, decisions would be respected and practical solutions sought to ensure anonymity and confidentiality, recognizing that “interactions between consenting and non-consenting individuals may still be relevant to the research” due to whole-class dynamics (BERA, 2018). No ethical issues arose; no parents contacted the school, class teacher, researcher, or community to withdraw or to inquire about processes, though they had been informed of that right.

A written parental consent form outlined in detail the study aim, procedures, ethics, lesson video recording, and the expectations and rights of both parties, including the parent/student right to withdraw without explanation at any time.

To ensure understanding of procedures and data handling (BERA, 2018), individual and small-group meetings (two to three parents) were organized to explain the study’s purpose, processes, and methods and to answer questions. Parents were informed about confidentiality, anonymity, voluntariness, open communication, and the possibility to withdraw at any time. They were told that lesson structure, goals, and activities would remain unchanged and aligned with the primary curriculum. Consent forms were provided and could be taken home for review and returned signed to the class teacher or researcher.

After parental consents were obtained, a cooperation agreement with the school was signed; leadership permission was granted; teachers and class teachers were informed; and support was requested for coordinating meetings with parents/guardians. The number of consents was assessed, and decisions were made on class suitability for participation.

Although parents signed consent, it was important that children also assented. The researcher informed children, in age-appropriate language, about the study aim and their right to decide freely to participate or withdraw.

To protect confidentiality and anonymity, the research does not disclose the institution’s name, city, or related information. Personal and school data were anonymized (student names removed; classes coded).

**As for the trustworthiness of our results,** we substantiate it through four principle criteria: (1) credibility; (2) dependability; (3) confirmability; and (4) transferability. These criteria have guided qualitative studies over recent decades. They unite the concepts of validity and reliability, and together characterize the research as “trustworthy” (Gibbs, 2018; Nassaji, 2020).

In addition to these criteria, the reliability of the study was confirmed by the applied method of researcher triangulation. As indicated in the research methodology literature, this method involves two (or more) researchers analyzing the same data set in order to obtain deeper and more reliable interpretations (Denzin, 1978, as cited in Carter et al., 2014; Bans-Akutey & Tiimub, 2021). The second author, although without access to the raw video recordings, reviewed the transcribed episodes derived from them and was able to agree with or challenge coding decisions, propose labels for themes and subthemes, and offer further observations. In this way, both researchers examined the same evidence and worked towards negotiated consensus, interpreting the data from two complementary perspectives and enhancing the precision and overall trustworthiness of the findings.

Credibility was achieved through the researcher’s perspective: the researcher had worked for several years as a teacher within the research setting. This enabled an in-depth familiarity with the sample, the context, and the specific nature of the work, and supported a nuanced understanding of the phenomenon analyzed.

Dependability was achieved through the appropriateness of the processes of data collection and analysis. The chosen methodology, and the theoretical and philosophical stances, fitted the phenomenon under analysis, the research context, and the aims. A micro-ethnographic approach was well-suited to analyzing such processes: it enabled repeated review of the entire course of events and the development of targeted interpretations. Following a social constructionist approach, the study explored how traits of emotional intelligence were developed during social interactions. It should also be noted that the phenomenon under analysis was broken down into smaller structural elements that were grounded in internationally recognized theory.

Confirmability was achieved through descriptive accounts of the activities conducted in physical education (PE) lessons. The lesson activities that were implemented align with the content and aims of the “Lithuanian Primary Education Curriculum”, and thus were clearly regulated and approved. The chosen approach allowed the researcher to delve into the data and analyze them from several vantage points; nevertheless, the final interpretation of the observed phenomenon was stated very clearly, distinguishing both the content of the social interaction in which it was manifested and the reasons for selecting that interaction, the action taking place within it, the forms of communication used, and so forth.

Transferability was achieved through the lens of theoretical premises for developing emotional intelligence and through empirical results of analysis of PE lesson episodes. Previous research has confirmed that the structure of emotional intelligence is the same for pupils with special educational needs (SEN) and for those without SEN. On the basis of these premises and the success of the empirical data, possibilities of the development of emotional intelligence in PE lessons were confirmed, and the same or similar social interactions and activities can be replicated in other contexts aiming to develop the emotional intelligence of pupils.

## 3. Results

The empirical data illustrating the factors that foster self-confidence and intrinsic motivation and their manifestations also reflect the academic context of PE lessons: instructional interactions between teacher and students; formulating learning goals; task types (warm-up, learning, conditioning, refinement); task instructions; opportunities to choose alternative (more difficult/easier) tasks; individualized support; and initiation of peer interactions.

Teacher–student interaction during instruction included both individual and group interactions: instructional communication (learning goals, task requirements); feedback on progress and results; and student (self-)assessment (results, effort, progress, behavior). As the analysis of the empirical data shows, the development of self-confidence and intrinsic motivation traits involves ongoing individual and group interactions between students and the teacher. Examining the content of the selected video episodes revealed shared teacher–student behavior patterns—recurrent interactions that differentially affect the observable expression of students’ emotions and behavior.

Thus, the academic context of physical education lessons helps to understand the emotional reactions of students with SEN when performing more difficult or easier tasks; and teacher–student interaction shows which factors promote the development of students’ self-confidence and intrinsic motivation.

By seeking study objectives and answers to research questions, based on empirical evidence (episodes selected per inclusion/exclusion criteria) and theoretical premises, we created a thematic map of the research data, illustrating (1) the academical context of self-confidence and intrinsic motivation education, (2) the factors of development of the studied EI traits, and (3) self-confidence and intrinsic motivation manifested in PE lessons (see Table 1).

Analysis of the empirical data identified four main themes: **(1) Constructive dialogic interaction; (2) Manifestation of the self-confidence trait; (3) Manifestation of the intrinsic-motivation trait; and (4) Student intrinsic self-motivation factors**. These themes demonstrate how the data analysis aligns with the study’s aim and elucidate the factors and manifestations of developing self-confidence and intrinsic-motivation traits among pupils with special educational needs (SEN) in physical education (PE) lessons.

**Figure 1:**
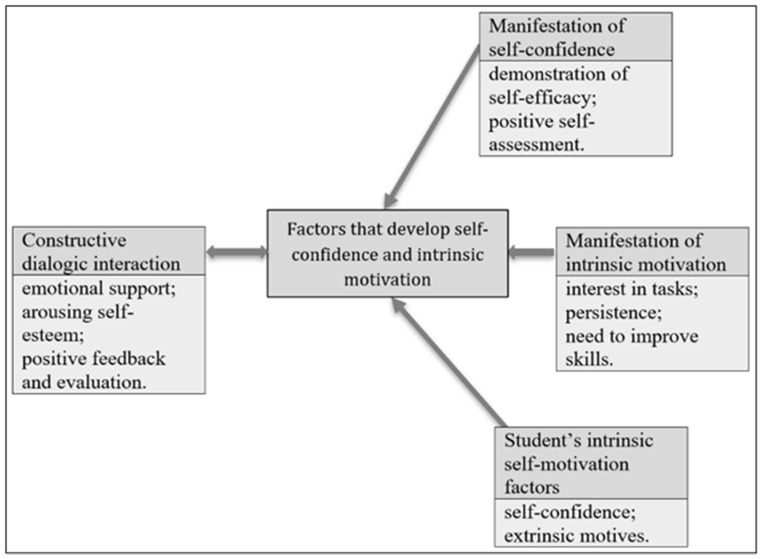
Relationships between developmental factors and manifestations of self-confidence and intrinsic-motivation traits.

The relationships among themes and subthemes are illustrated in Figure 1.

The first theme, **Constructive dialogic interaction**, is a unifying theme describing the developmental factors for both emotional intelligence traits—self-confidence and intrinsic motivation. The term “dialogic” underscores the individualized, student-centered nature of this interaction. The term “constructive” denotes its positive directionality: emotional support for the student, activation of self-esteem, and positive feedback, all aimed at helping the student develop self-confidence and intrinsic motivation.

Two themes *Constructive dialogic interaction* and *Student intrinsic self-motivation factors* capture contextual and individual factors in the development of self-confidence and intrinsic motivation. The two other themes *Manifestation of self-confidence* and *Manifestation of intrinsic motivation* describe the indicators of self-confidence and intrinsic motivation in physical education lessons.

A synthesis of the relationships between the developmental factors and the manifestations of self-confidence and intrinsic motivation is presented in the Discussion section of this research.

Examples illustrating the themes and subthemes are presented with episode codes.

### 3.1. Factors in Developing Self-Confidence: Constructive Dialogic Interaction

Within the theme *Constructive dialogic interaction*, the subthemes that describe the factors for developing the self-confidence trait are **emotional support**, (to perform the task, belief in the student’s efficacy, highlighting the student’s strengths); **arousing self-esteem** (prompting the student to show their abilities and to complete the task successfully); and **positive feedback and appraisal** (positive evaluation of the student’s effort and positive appraisal of results and progress).

In some cases, when students recognize that they have achieved an acceptable result, they themselves initiate interaction with the teacher to confirm the outcome obtained. It can be assumed that interaction with the teacher in such cases helps students to perceive their capability and internalize self-confidence. The teacher helps students self-assess the result, encourages and praises them, and evaluates their achievements.

When students show effort to achieve an acceptable result but fail to attain it, they perceive this as an unacceptable outcome, which they communicate by withdrawing from the activity and expressing negative emotions. In such cases, the teacher applies methods of **emotional support**—helping students develop self-confidence by encouraging, praising, and recognizing the effort demonstrated. This is illustrated, for example, in Episode BGHPS0310 1.0.

Episode BGHPS0310 1.0. Context: teacher’s constructive dialogic interaction with the student—**positive feedback and evaluation of effort**: the teacher initiates individual interaction with VB1 and evaluates the student’s effort and engagement in the lesson; and **arousing of self-esteem**, expressed as belief in the student’s efficacy: the teacher encourages the student by commenting on his capability to handle such tasks and prompts him to regulate negative emotions and complete the task.

**Manifestation of self-confidence—self-efficacy and willingness to perform the task; reflective awareness of one’s capabilities**: VB1 regulates his negative emotions and nonverbally demonstrates acceptance of the teacher’s encouragement and awareness of his capabilities.

In this situation, **the teacher’s constructive interaction—emotional support** (encouragement; expressed belief in the student’s efficacy)—helps the student maintain a positive self-view, fosters a sense of self-efficacy, and supports development of the self-confidence trait.

### 3.2. Manifestation of the Self-Confidence Trait: Demonstrating and Reflecting Self-Efficacy

The episodes analyzed below are illustrative examples that constitute the content of the themes and subthemes identified in the study. In each example, the episode context is presented in terms of the lesson’s learning objective (the pupils’ goal for the lesson), the situation (what is occurring in the episode), and the reaction (the pupils’ response to the situation).

Analysis of the empirical data revealed the theme of students’ **manifestation of self-confidence**. The subthemes were **demonstration of self-efficacy**, (a clearly expressed desire to perform the task; awareness of one’s strengths; active engagement in task performance and positive emotions while performing the task and after success); and **positive self-assessment** (reflective awareness of one’s capabilities or strengths and positive evaluation of both the effort invested and the result achieved).

Students’ manifestation of self-confidence is enabled by creating **conditions** to experience subjective success. Such conditions are created by identifying students’ anxiety and lack of self-confidence before and/or during task execution, when students appraise their own capability. **Preconditions** for developing the self-confidence trait are established by individualizing the educational content (tasks) so that students can experience success and attain acceptable results.

In some cases, the experience of success is achieved by incorporating additional individualized assistance from classmates in performing the task, or by allowing the student to repeat the task with encouragement and additional tailored guidance and prompts—for example, in Episode PGXPS0004 1.0.

Episode PGXPS0004 1.0. Context: students perform a complex body-weight movement hesitantly.

**Constructive dialogic interaction—emotional support, encouragement, and positive feedback**: the teacher praises and encourages students to attempt the next trial more effectively, also providing individualized assistance to each student.

**Manifestation of self-confidence**—demonstration of self-efficacy, **clearly expressed desire to perform the task**: students, without directly interacting with the teacher, make their attempts; after completing the task, they return to the line to repeat attempts; positive emotions after success: students experience success and express positive emotions. 

Manifestations of students’ self-confidence were also observed during conditioning tasks, for example, in Episode PGHPS0094 1.0.

Episode PGHPS0094 1.0. Context: situation—providing a choice of alternative (more/less difficult) tasks: the teacher proposes a more complex exercise; individualized support: the teacher assigns individualized tasks to each student.

**Manifestation of self-confidence—demonstration of self-efficacy, clearly expressed desire to perform the task**: all students express a willingness to perform the exercise.

**Constructive dialogic interaction—positive feedback; appraisal of progress**: in the teacher’s view, JK11 demonstrates the exercise very well and is praised for progress, as an advanced student, JK11, is selected to demonstrate the exercise.

**Manifestation of self-confidence—positive self-assessment; reflective awareness of one’s capabilities**: the student feels recognized and reflects on personal abilities.

Students’ self-confidence is also developed during group interactions with the teacher while performing learning, refinement, and conditioning tasks, as well as during breaks—for example, in refinement tasks in Episode PGHPS0356 1.0.

Episode PGHPS0356 1.0. Context: situation—the teacher creates a context in which students display awareness of individual success and progress made.

**Constructive dialogic interaction—positive feedback and evaluation of effort**: for their efforts and for avoiding major mistakes, the teacher praises individual students (VB11, VB12, VB8, VB6, VB7); the teacher encourages continued effort on the next task and helps students (self-)assess their progress.

**Manifestation of self-confidence—demonstration of self-efficacy; clearly expressed desire to perform the task**: the praised students do not enter direct interaction with the teacher; they run to fetch their equipment; **positive emotions during performance and after success**: they display positive emotions and a sense of individual success.

The self-confidence trait is also cultivated during peer interactions in which the teacher is an active participant, for example, when students perform movement/skill refinement tasks.

Episode PGHPS0148 1.1. Context: learning objective—refine skills for competing in active games.

Situation—student NZ9 fails on the first attempt.

**Constructive dialogic interaction; individualized support**: the teacher initiates interaction with NZ9, proposes and creates conditions to attempt the task again individually; **emotional support**: encouragement; the teacher repeatedly encourages NZ9 to perform the task and reminds her of the requirements; **belief in the student’s efficacy**: the teacher encourages NZ9 to form an appropriate perception of her effectiveness.

Peer interaction: after experiencing success in performing the task with NZ10, the two students attempt it again together.

**Manifestation of self-confidence**—demonstration of self-efficacy; **clearly expressed desire to perform the task:** in interaction with NZ10, the student feels more confident; positive emotions after success: after completing the task, NZ9 experiences success, expresses positive emotions, and returns to the rest of the group.

Manifestations of students’ self-confidence were also observed while overcoming challenges. It was found that students develop self-confidence in two ways, depending on the outcome achieved. When students demonstrated an acceptable result, the teacher praised them and encouraged them to positively self-assess the result. In cases of failure, the teacher positively evaluated the effort shown, identified strengths, and provided encouragement—for example, Episode ĮGHPS0196 1.0.

Episode ĮGHPS0196 1.0. Context: learning objective—refine two-handed set-shot technique in basketball. Situation—during warm-up exercises, student NZ10 initiates interaction with the teacher, expresses negative emotions, and reports the task’s difficulty.

**Constructive dialogic interaction—emotional support, encouragement, belief in the student’s efficacy**: the teacher evaluates NZ10’s capability and prior success with this task and encourages her by explicitly naming her abilities to perform it.

**Manifestation of self-confidence—demonstrated awareness of self-efficacy**: the teacher’s encouragement helps NZ10 positively appraise her capability.

**Constructive dialogic interaction—positive feedback; appraisal of progress**: after NZ10 successfully completes the task, the teacher praises her and again emphasizes her capability to perform the task.

**Manifestation of self-confidence—positive evaluation of the result**: NZ10 verbally acknowledges successful task completion and continues with the lesson.

In situations where insufficient self-confidence is observed after encountering difficulties, the teacher’s constructive dialogic interaction and positive feedback are crucial. Students do not always perceive or appraise their capability while performing a task. By evaluating the difficulties overcome, the progress demonstrated, and the result achieved, the teacher helps students develop the self-confidence trait.

### 3.3. Development of Intrinsic Motivation: Constructive Dialogic Interaction and Students’ Intrinsic Motivation

Constructive dialogic interaction aimed at fostering intrinsic motivation includes the subthemes **emotional support** (belief in the student’s efficacy and highlighting the student’s strengths); **arousing of self-esteem** (encouraging the student to show their abilities and to complete the task successfully); and **positive feedback** (evaluating the student’s effort, result, and/or progress).

Within the theme **Student Intrinsic motivation factors**, the subthemes include **self-confidence** (student’s prior successful experience and positive emotions experienced) and **possible latent external motives** (desire to receive positive evaluation and to enhance one’s image.

For example, Episode PGHVM0123 1.0 illustrates a situation in which self-esteem is aroused by creating an opportunity to demonstrate competence by choosing a more difficult alternative.

Episode PGHVM0123 1.0. Context: Situation—students perform the usual exercises in sequence; it is student JK4’s turn.

**Constructive dialogic interaction—positive feedback; appraisal of progress**: the teacher evaluates the progress made by JK4; **emotional support—expressed belief in the student’s efficacy**.

Manifestation of self-confidence—positive emotions: JK4 nonverbally expresses positive emotions.

**Constructive dialogic interaction—providing a choice of alternative (more/less difficult) tasks**: to encourage the student, the teacher allows her to choose between a more difficult and an easier version of the exercise.

**Intrinsic motivation factors—self-confidence: prior successful experience; active engagement in task performance; latent external motive**—the desire to demonstrate competence: the student appraises her capability and accepts the teacher’s indirect challenge, choosing the more difficult version.

Episode BGHVM0009 1.0 illustrates a situation in which **self-esteem is aroused** by creating an opportunity to demonstrate leadership, taking a principal role in a game.

Episode BGHVM0009 1.0. Context: situation—the teacher initiates interaction with the students and proposes selecting a new main game leader.

**Manifestation of intrinsic motivation—interest in the task**: students VB8, VB2, VB12, VB11, VB10, VB5, and VB4 support the teacher’s proposal and express a strong desire to take on the main role.

Another episode, ĮGHVM0343 1.0, illustrates a situation in which **self-esteem is aroused** by creating an opportunity to demonstrate superiority over peers in a competitive context.

Episode ĮGHVM0343 1.0. Context: situation—during a familiar outdoor warm-up, student JK2 initiates interaction with peers, claiming physical superiority. Students JK9, JK8, and JK12 verbally dispute this.

**Intrinsic motivation development factors—arousing of self-esteem through constructive dialogic interaction**: the teacher initiates interaction, proposes a challenge, and creates conditions for students to choose a task that allows them to demonstrate potential physical superiority.

**Manifestation of intrinsic motivation—interest in the task**: JK4 nonverbally expresses positive emotions and accepts the teacher’s challenge, choosing the more difficult exercise variant.

This example shows how intrinsic motivation can be stimulated by a **latent external motive—a desire to showcase abilities—as well as by self-confidence**, i.e., the desire to establish superiority over peers, which is supported by prior successful experience and positive emotions following success.

### 3.4. Manifestation of the Intrinsic-Motivation Trait: Interest, Persistence, and Improvement

The manifestation of intrinsic motivation is revealed through the subthemes: **interest in tasks** (active engagement in task performance and choosing challenging tasks); **persistence when performing challenging tasks** (efforts to perform a difficult task well and resolve and perseverance in overcoming difficulties); and **need for skill improvement** (demonstrated desire to develop one’s abilities and achieve a better results).

Indicators of intrinsic motivation—interest and desire to perform the task—are illustrated by Episode PGHVM0240 1.0.

Episode PGHVM0240 1.0. Context: situation—students are preparing to perform the lesson task; student VB12, who has been excused from the task, initiates interaction with the teacher.

**Manifestation of intrinsic motivation—interest and desire to perform the task**: VB12 asks the teacher to allow participation in the task.

**Constructive dialogic interaction—the teacher creates conditions** for VB12 to participate in a low-intensity task **by proposing additional arrangements**.

**Manifestation of intrinsic motivation—interest and desire to perform the task**: VB12 verbally expresses positive emotions and proceeds to prepare for the task.

Another indicator—**the need for skill improvement**—is illustrated by Episode PGHVM0354 1.0.

Episode PGHVM0354 1.0. Context: situation—student JK11 has performed his individualized task exceptionally well on several occasions. The teacher signals the end of the group task and announces a break.

**Manifestation of intrinsic motivation—desire to improve skills**: JK11 initiates interaction with the teacher, expressing a desire to repeat the task once more. Having applied his abilities effectively, he feels satisfied with having performed the task well and wants to repeat it to relive this feeling.

Episode PGHVM0303 1.0 illustrates two indicators of intrinsic motivation: interest and the need for skill improvement.

Episode PGHVM0303 1.0. Context: situation—to activate students, the teacher informs them that a task they enjoyed in the previous lesson will be performed.

**Manifestation of intrinsic motivation—interest and desire to perform and improve**: JK9, JK3, JK4, JK5, JK8, JK11, and JK10 express positive emotions and verbally confirm their desire to perform the task.

When performing refinement tasks, intrinsic motivation is nurtured by setting the goal of **completing a challenging task**—for example, Episode ĮGXPS0311 1.0.

Episode ĮGXPS0311 1.0. Context: situation—student JK1, while performing a difficult warm-up task, initiates interaction with the teacher seeking individualized assistance.

**Constructive dialogic interaction: the teacher draws on other students as positive examples, encourages modeling on their performance, and praises demonstrated progress; encouragement to perform the task; belief in the student’s efficacy; highlighting strengths; activation of self-esteem**: the teacher encourages JK1 to reflect by comparing the difficulties experienced in the previous lesson with the performance demonstrated in the current lesson.

**Manifestation of intrinsic motivation—persistence, effort to perform a difficult task well, determination to overcome difficulties**: JK1 nonverbally confirms understanding of the teacher’s statements and, showing positive emotions, continues the activity and strives to complete the task.

Episode ĮGXPS0311 1.0 illustrates how constructive dialogic interaction arouses self-esteem and encourages the student to showcase abilities. Here, intrinsic motivation is indicated by the student’s persistence in performing a challenging task, effort to perform it well, and resolve to overcome difficulties. **A latent external motive—to demonstrate one’s abilities**—may also be present.

## 4. Discussion

The study is grounded in trait emotional intelligence theory, which explains an individual’s perceived ability to recognize, process, and manage emotions (Salovey & Mayer, 1990). Self-confidence and intrinsic motivation constitute only part of the multidimensional components of the trait emotional intelligence construct (Petrides et al., 2016; Petrides & Mavroveli, 2018). In this study, self-confidence is understood as one’s positive self-esteem, an opinion about one’s abilities to overcome challenges and be successful (Petrides & Furnham, 2001; Petrides et al., 2016; Petrides & Mavroveli, 2018; Bhat & Arumugam, 2021; Manikandan, 2015; Vărăşteanu & Iftime, 2013; Sharma & Sharma, 2025), and intrinsic motivation—as an individual’s interest in, engagement with, and satisfaction derived from an activity (Ryan & Deci, 2020; Bandhu et al., 2024). Intrinsic motivation promotes greater engagement, creativity, and well-being (Bandhu et al., 2024), and as our and other studies (Arias et al., 2022; Bandhu et al., 2024; Wiliyanto et al., 2025, etc.) illustrate, may be associated with self-confidence or other EI traits. This allows us to explore the development of self-confidence and intrinsic motivation as two related traits of emotional intelligence.

Many previous studies have identified the characteristics of trait emotional intelligence, but have not thoroughly examined the subtleties of the process of developing self-confidence and intrinsic motivation in students with SEN.

The aim of this study was to analyze the factors of developing and manifestations of self-confidence and intrinsic motivation in pupils with special educational needs (SEN) during physical education (PE) lessons. Employing a micro-ethnographic methodology, video segments (episodes) of PE lessons were analyzed, focusing on teacher—pupil interactions and on pupils’ behavioral and emotional responses as empirical indicators of their manifestation of self-confidence and intrinsic motivation.

### 4.1. Different Tasks as Environments to Foster Self-Confidence and Intrinsic-Motivation Traits

Analysis of the empirical data enables an answer to **RQ1**: In which activities during physical education lessons do students develop self-confidence and intrinsic-motivation traits?

A note from the researcher reflexivity: *One intention of this research was to identify the activities through which pupils’ emotional intelligence traits are developed. However, analysis of the data indicated that it is not the activities per se but the social interactions that are fundamental to the development of these traits, which, in my view, enrich the results and form an integral part of the study’s central argument. The physical activity itself became a secondary or tertiary variable.*

Students develop self-confidence and intrinsic motivation across a variety of physical education activities selected according to instructional aims. In this study, task demands varied with the components of the physical education program implemented in lessons, the learning objectives set for students, and the types of tasks (warm-up, learning, conditioning, refinement tasks). Students performed tasks that for some of them were easy or difficult to overcome depending on students’ individual abilities and capacity. Some participating students exhibited inattention, emotional lability, and difficulties in emotion regulation. With more difficult tasks, students sometimes showed self-doubt even before performing them. Self-confidence was developed when encouraging them to overcome challenges, introducing alternative equipment, and otherwise individualizing instruction. After successful completion—especially when preceded by self-doubt—students, with the teacher’s help, reappraised their capability. When acceptable results were achieved, students sometimes sought teacher confirmation and positive evaluation of their outcomes. The affirmation of the student’s self-efficacy elicited positive emotions, strengthened self-confidence, and motivated them to overcome further challenges, seeking progress.

In fostering intrinsic motivation in physical education lessons, the variety and dynamics of tasks, their intensity and appeal, individualized support with manageable challenges, and emotional support enabling success for students of varying ability are key to creating opportunities to enjoy task performance.

Physical education (PE) lessons are particularly well-suited to develop emotional intelligence traits because of versatility activities and the emotions pupils experience during the learning process. Marzec’s (2017) research showed that physical activity can increase self-esteem and believing in one’s own strength, thus supporting positive social development and preventing risky behaviors. Developing self-confidence is particularly relevant for students with SEN, who experience a variety of academic and social developmental difficulties.

Thus, in response to research question 1, we argue that students can develop traits of self-confidence and intrinsic motivation in various physical education lesson activities by completing various types of tasks. Our study data revealed that the PE tasks themselves are not the principal factor in developing pupils’ self-confidence and intrinsic-motivation traits. Rather, the key lies in creating conditions that enable the manifestation of these EI traits, i.e., in the teacher’s constructive dialogic interaction methods. Such methods can be designed and adapted across different activities, various academic contexts (e.g., physical activity education aims, program, etc.), and depending on pupils’ special educational needs. These insights align with the findings of Maclellan (2014) and Bada and Olusegun (2015), namely that pupils’ emotional intelligence can be cultivated when the teacher acts as a mediator and, through appropriate social interaction, helps pupils to develop their emotional capacities.

The results partly corroborate the conclusions of Fernandes et al. (2024) and Wang et al. (2025) that engagement in physical activity promotes the development of emotional intelligence. Although this study did not directly measure emotional intelligence traits, based on the empirical data, themes and subthemes were constructed to reveal processes of developing emotional intelligence traits within physical education lessons.

### 4.2. The Teacher’s Role in Empowering Students to Develop Self-Confidence and Intrinsic-Motivation Traits

As a result of the reflexive thematic analysis of empirical data, two themes were identified—*Constructive dialogic interaction* and *Student Intrinsic motivation factors*—and this data helped to answer **RQ2:** Which actions by teachers help students develop self-confidence and intrinsic-motivation traits?

**Constructive dialogic interaction**. The findings of study revealed that the teacher’s constructive feedback and targeted attention to emotional difficulties of pupils with SEN are the key factors that create opportunities to develop self-confidence and intrinsic-motivation traits. Constructive dialogic interaction is the unifying theme and the key factor promoting the manifestation of both self-confidence and intrinsic motivation traits (see Figure 1). Teacher’s emotional support (encouragement; belief in the student’s efficacy; highlighting strengths), activation of self-esteem (prompting students to show their abilities and to complete tasks successfully), and positive feedback (positive evaluation of effort, results, and progress) stimulate pupils’ self-confidence; self-confidence, together with with persistent positive interaction, stimulates pupils’ motivation to make efforts to perform tasks better. All indicators of constructive dialogic interaction are interrelated, and each function is a factor in developing self-confidence and intrinsic motivation.

Maclellan (2014) also noted the importance of learner–teacher interactions for promoting self-confidence. Important for our study is Maclellan’s (2014) conclusion that teachers can actively help students overcome stereotypical beliefs about insurmountable difficulties that lead to their loss of self-confidence. Self-confidence and intrinsic motivation are most effectively developed through individual teacher–student interactions and constructive dialogic exchange. Prior findings corroborate this: students can gain self-confidence in classrooms that provide positive comments and a supportive learning environment (Venkataraman, 2021). Constructive feedback and encouragement can strengthen students’ self-confidence (T. Jansen et al., 2025).

Our research supports these ideas by highlighting that increasing self-esteem as a factor for developing both traits manifested in two ways. First, self-esteem is linked to perceived self-efficacy and self-confidence. Externally, such self-esteem becomes visible through students’ efforts to tackle challenging tasks and through peer interactions such as competition and the desire to appear superior to classmates and to earn positive evaluation (external rewards). Second, self-esteem also prompts students to improve skills and achieve better results (an internal motive)—goals that require self-confidence and belief in one’s abilities. Thus, activating self-esteem promotes the development of both self-confidence and intrinsic motivation. Although this study did not examine the impact of the identified pedagogical methods on trait emotional intelligence, it placed considerable emphasis on the teacher’s role in their development. Of course, every teacher may conceptualize the development of trait emotional intelligence differently; nonetheless, this study identifies a set of guiding principles for practice that can inform teaching and development of the mentioned traits.

For students with severe special educational needs, learning processes in physical education tasks are lengthy, and progress is not always linear. The teacher continuously observes, engages in reciprocal reflection, evaluates, and helps students self-assess their progress, results, strengths, and areas for improvement. Constructive dialogic interaction during every physical education activity creates opportunities not only to develop physical skills, but also to continually monitor personal and classmates’ progress, appraise one’s capability and abilities, gain self-confidence, and build motivation to achieve better results.

To develop students’ self-confidence, teachers consistently applied positive feedback about strengths and areas for improvement and encouragement that emphasized students’ capacities rather than limitations, thereby dispelling self-doubt. Positive feedback is valuable even in situations of doubt or low self-confidence.

**Factors in developing intrinsic motivation**. Based on the empirical data analysis, *Student intrinsic motivation factors* were related to their self-confidence and to latent indicators such as prior successful experience. Indicators of intrinsic motivation were pupils’ positive emotions during tasks performed, and their desire to repeat tasks again and to re-experience the joy of success. Besides it, external motives were identified, with indicators such as the desire to receive positive evaluation and to enhance one’s image (e.g., demonstrate superiority over peers)—showing that external rewards also matter in the formation of intrinsic motivation.

Hence, intrinsic motivation factors are linked both to teacher constructive dialogic interaction with pupils and to pupils’ self-confidence (see Figure 1).

### 4.3. Manifestion of Pupils Self-Confidence and Intrinsic-Motivation Traits

The exploration data on pupils’ emotional and behavioral reactions during teacher-pupils interaction also revealed the answer to **RQ3**: How are students’ self-confidence and intrinsic-motivation traits manifested in physical education lessons?

The themes and subthemes describing the latent manifestation of self-confidence and intrinsic motivation were constructed on the basis of observable emotional reactions, actions, and behaviors. The study analyzed pupils’ emotional responses that imply difficulties associated with limited self-confidence or motivation traits. Examination of the data showed that such responses were frequent in this sample and were highly salient when pupils evaluated their performance, irrespective of whether the outcome was positive or negative. These observations are partly corroborated by findings from Mavroveli and Sánchez-Ruiz (2011) and Abo Elella et al. (2017), which reported that pupils with special educational needs (SEN) score lower on trait emotional intelligence measures than their neurotypical peers.

On the other hand, although those and many other studies have associated children with SEN with maladaptive behavior, low self-esteem, lack of social skills, and other related social and emotional difficulties, our study revealed that under supportive conditions, such as constructive dialogic interaction from the teacher, these children tend to demonstrate self-confidence, which also increases their intrinsic motivation to achieve. However, further research should analyze the stability of self-confidence and intrinsic motivation in these students.

Our research on self-confidence and intrinsic motivation manifested within the research scope.

**Manifestation of the self-confidence trait:** The theme *Constructive dialogic interaction* is reciprocally related to *Manifestation of self-confidence* (see Figure 1), indicated by *demonstrated self-efficacy* (a clearly expressed desire to perform the task; active engagement; positive emotions during performance and after success) and positive self-assessment (reflective awareness of capabilities or strengths; positive appraisal of effort and results).

A section from the researcher’s self-reflection: *It was observed that participating students exhibited lower self-confidence. When preparing to perform complex or more difficult tasks, they tended to avoid certain tasks or perform them hesitantly until the teacher began to encourage them intensively. Low self-confidence was also indicated by students’ lack of understanding of how and why they were capable of performing particular tasks. They recognized their capability only after achieving a specific result and appraising it themselves or receiving teacher appraisal; self-confidence then emerged but was not sustained.*

Students generally participated willingly in physical education lessons, but more complex, challenging tasks that required greater effort likely induced anxiety and suppressed both their self-confidence and motivation. Although there is no evidence of a universal relationship between emotional states and PE outcomes, some studies confirm that self-confidence and negative emotional states such as anxiety are moderately to strongly negatively correlated (Jekauc et al., 2021). Self-confidence can be considered to be the opposite of such negative emotions and our research supports this idea. Students experienced both success and difficulty during lessons. Rules and requirements were not always followed smoothly, and some episodes of inappropriate behavior occurred. In such cases, the teacher helped students understand that mistakes are part of learning, reminding them of rules for appropriate behavior. This supported the development of self-confidence and stimulated intrinsic motivation to overcome difficulties.

In the dynamic context of physical education lessons, students experienced various emotions, expressed both verbally and through body language and facial expressions. The selected episodes revealed emotional cycles: uncertainty before tasks; active participation and engagement; joy and positive self-evaluation after success; recognition of progress; and resolve to overcome challenges. Analysis of these shifts helps identify manifestations of self-confidence and intrinsic motivation across situations: desire to perform tasks; efforts to surmount challenges; and understanding of one’s capabilities as the teacher provides constructive information about their achievements.

Our data suggest that students’ anxiety was reduced by constructive dialogic interaction, whereby the teacher created opportunities to experience success and joy in task performance. Ongoing constructive dialog, emotional support, and encouragement—evaluating effort, progress, and other aspects—positively influenced the development of self-confidence. Students experienced joy when they successfully performed tasks by leveraging strengths, overcame challenges, and positively self-assessed personal progress in individual and group interactions with the teacher.

A note from the researcher’s reflexivity: *Looking back at how I supported my pupils in overcoming difficulties during the study, I recognize that I tended to rely on a more traditional teacher perspective rather than approaching my role creatively and in a modern way to help the pupil. At the time, my understanding of activity organization was oriented towards outcomes rather than process. Although pupils perceived me as an older friend, I noticed that I often forgot this relational stance and focused on helping them learn, do, and complete tasks—rather than enjoy, delight in, or truly engage with them. As a result, pupils experienced not only positive but also negative emotions—both in their interactions with me (rather than with peers) and in response to the processes of task performance. While the expression of negative emotions is analytically useful for interpreting the data and results, these reactions could have been mitigated had I adopted a different teaching philosophy or professional stance. Teachers, like pupils, are diverse and characterized by individual differences.*

**Manifestation of the intrinsic-motivation trait**. Indicators of intrinsic motivation: interest in tasks (active engagement; choosing challenging tasks), persistence in performing challenging tasks (efforts to perform difficult tasks well; resolve to overcome difficulties), and need for skill improvement (demonstrated desire to improve abilities and achieve better results).

Pupils’ intrinsic motivation manifested in resolve and perseverance to achieve not only acceptable but higher results, e.g., choosing more difficult tasks from among alternatives proposed by the teacher.

The data analysis suggests that students’ emotional states influence their motivation to engage with tasks and challenges and are indirectly linked to their achievements and outcomes. In physical education lessons, these traits manifest through students’ emotional reactions—before tasks, after success or failure, and when recognizing individual strengths and capability in interaction with teachers and peers. Research participants showed a strong desire for positive evaluation and were sensitive to the teacher’s comments about their abilities. Consequently, the video episodes frequently observed teacher feedback—constructive dialogic interaction ranging from simple comments or advice to encouragement and positive appraisal of results or progress. The present findings corroborate other researchers: external rewards can strengthen intrinsic motivation and engagement when delivered in ways that support autonomy and competence (Bandhu et al., 2024). Nevertheless, over-emphasis on external rewards can undermine intrinsic motivation; thus, a balance between external rewards and the fostering of intrinsic motivation is essential (Bandhu et al., 2024). So, a necessary condition is to combine information about actual results with encouragement regarding expected individual achievements (Maclellan, 2014).

The empirical data suggest that a student’s self-confidence likely stimulates intrinsic motivation and functions as a factor in its development. One indicator of both self-confidence and intrinsic motivation is *choosing more difficult tasks from among alternatives proposed by the teacher*. Individuals with higher self-efficacy set themselves higher goals and invest greater effort to achieve them (Bandura, 2012). Accordingly, students in our study who chose more difficult tasks demonstrated self-confidence, self-efficacy, and *intrinsic motivation (resolve and perseverance) to achieve not only acceptable but higher results*. Other research confirms that offering meaningful choices, providing successful learning experiences, and cultivating supportive relationships strengthen intrinsic motivation and engagement—underscoring a balanced approach that acknowledges both external and internal motivators (Bandhu et al., 2024).

Many of the analyzed lesson episodes reflect a strengths-based practice in which the teacher, through constructive dialogic interaction, highlights students’ abilities—even when they do not always succeed at tasks. The empirical data indicate that noticing strengths promotes both self-confidence and intrinsic motivation, improves the overall emotional climate, and reduces anxiety about task difficulty. This aligns with prior research showing that strengths-based strategies enhance students’ perceptions of competence, intrinsic motivation, and intentions to improve their abilities (Niemiec & Pearce, 2021), including among students with special educational needs (Shogren et al., 2014).

The study found that the teacher’s constructive dialogic interaction fostered not only students’ self-confidence but also their intrinsic motivation and the manifestation of these traits. From a social-cognitive perspective, constructive dialogic interaction—especially emotional support and positive feedback—stimulated students’ self-awareness (recognizing strengths and weaknesses) and their corresponding emotional and behavioral responses. In other words, constructive dialogic interaction builds self-confidence, which in turn activates intrinsic motivation to improve abilities. This is supported by other studies—for example, positive feedback can strengthen athletes’ self-confidence and thereby their motivation (Sari et al., 2015).

From a constructivist, learner-centered perspective, feedback that helps students understand what is expected of them and how their efforts improve their performance fosters emotional and cognitive engagement (Heitink et al., 2016). A review by Heitink et al. (2016) showed that targeted feedback can have a substantial impact on learning motivation. Research on social-constructivist feedback confirms the importance of reciprocal teacher–student interaction to develop feedback processes so that students understand evaluative comments and use them to improve (Carless & Winstone, 2023). Although feedback can elicit strong emotions and threaten self-esteem, emotions are a natural part of feedback processes (Carless & Winstone, 2023); constructive responses balance negative and positive aspects (Bourne & Foster, 2024) and integrate both cognitive and socio-affective support strategies (Xu & Carless, 2017).

The importance of contextual factors and the dynamic interaction between individuals and their environment is emphasized both in social constructivism (Berger & Luckmann, 1991) and social-cognitive theory (Bandhu et al., 2024). This perspective recognizes that motivational factors are not only individual (e.g., self-efficacy, self-confidence, self-esteem) but also social and environmental (Bandhu et al., 2024).

Constructive dialogic interaction occurs when students demonstrate positive results, effort and self-confidence, or uncertainty about their abilities. By responding to manifestations of (un)confidence—positive or negative emotions, active engagement, or task avoidance—the teacher initiates interaction and builds the development of the self-confidence trait by providing emotional support (encouragement), continually positively appraising progress and outcomes, and helping students identify their strengths and self-assess capability, effort, results, and success.

A note from the researcher’s reflexivity: *Based on this study, I believe that answers have been found, at least in part, to the problematic questions identified in the literature even before this study. However, a teacher with a different philosophy or set of assumptions might reveal other results or extend those presented here. In my own practice, guided by these findings, I have begun to conduct some activities differently—more purposefully and more creatively.*

### 4.4. Strenghts and Limitations

Strengths of the research: The lead author’s professional experience and direct participation in the field motivated the choice of micro-ethnographic methodology. Direct involvement enabled the collection of abundant authentic data through ongoing participation and contact with study participants. The author continually reflected on the potential influence of his roles as a researcher and teacher on interpretive subjectivity. Nonetheless, this subjectivity did not affect theme generation: empirical data were analyzed inductively, and interpretive insights were subsequently substantiated by trait emotional intelligence theory and reflexive thematic analysis. Reflexive TA allows data-driven analysis while leveraging existing theory to deepen interpretation (Braun et al., 2023). In interpreting episodes, reflexive TA treats the teacher–researcher’s subjectivity as a strength, insofar as reflection is a natural process that enriches the data.

This study supplements research on emotional intelligence traits with new empirical data about how self-confidence and intrinsic motivation are cultivated in children with severe special educational needs through interactions in the context of physical education lessons. The empirical results and conclusions may be useful in advancing research ideas on developing emotional intelligence traits and in expanding teachers’ knowledge about practical specificities of fostering self-confidence and motivation in students with special educational needs, especially in the contexts of inclusive education. The empirical results reflect universal methods for developing emotional intelligence traits in students with SEN and can be transferred to other educational contexts.

Some limitations stem from the chosen method of video recording activities during lessons. On the one hand, filming enabled the collection of authentic data relevant to the study. On the other hand, analyzing physical education episodes was challenging due to the dynamic and noisy environment. During video analysis, technical constraints sometimes impeded audibility of verbal interactions or made them inaudible; therefore, the content of students’ verbal reactions and their verbal interactions with the teacher and peers was not interpreted.

Another limitation is that changes in the traits of self-confidence and motivation during the instructional process were not measured. This means that the effectiveness of revealed methods and strategies are unknown and only partly supported by the researcher’s reflexivity on pupil progress.

### 4.5. Future Directions

The limitations of this research call for further studies employing complementary methods. It would be valuable to determine the effectiveness of the identified methods and strategies through quantitative studies. It would also be worthwhile to conduct similar research with larger samples to enhance the reliability of the results. In line with the principles of inclusive education, such studies should be undertaken specifically within these contexts; in these settings, it would likewise be useful to measure the effectiveness of the identified methods and strategies.

In line with the trait emotional intelligence framework, similar studies could be conducted on the other traits within this structure. As noted in the study, pupils with SEN tend to exhibit lower trait emotional intelligence scores; therefore, these pupils are likely to require development not only of self-confidence and intrinsic-motivation traits, but also of related traits such as emotion perception, emotion regulation, emotion expression, low impulsivity, peer relations, affective disposition, self-esteem, and self-motivation.

### 4.6. Implications

Having evaluated the study’s results and conclusions—particularly the characteristics of teacher–pupil communication and their added significance—the findings may be valuable both for the preparation of novice specialists and for the competence development of experienced teachers. The methods and strategies identified in the study do not require additional equipment or specialized programs; rather, they generate new theoretical knowledge and practical skills regarding how the content of social interactions can support the development of pupils’ emotional intelligence traits. It is also worth noting that physical education (PE) lessons are compulsory for almost all pupils; accordingly, the teacher’s practical ability to cultivate these traits concurrently is especially valuable. Upon successful implication of these findings, PE lessons might gain additional functionality and importance.

As demonstrated in this article, pupils with special educational needs often lack traits of emotional intelligence, including two core traits—self-confidence and intrinsic motivation. Physical education lessons provide an excellent educational environment for cultivating these traits, and the methods and strategies identified in the study do not require changes to PE lesson content or special programs.

For example, if a teacher identifies activities in which pupils are more likely to demonstrate self-efficacy (i.e., manifestations of self-confidence), these can be incorporated more frequently, alongside the methods of constructive dialogic interaction (emotional support, or positive feedback). Such a strategy both centers activities acceptable to pupils and fulfills the aims of PE lessons, while developing traits of pupils’ emotional intelligence—in this case, self-confidence.

Another example can be given that concerns a teacher aiming to develop pupils’ intrinsic-motivation trait. The study revealed that this trait manifests through pupils’ interest in tasks. A teacher who understands which tasks create the conditions for pupils to demonstrate such interest can apply them more frequently. When pupils display this interest, the teacher can employ constructive dialogic-interaction methods—for instance, enhancing self-esteem—thereby fostering pupils’ satisfaction with the activity without external reward and, in turn, cultivating their intrinsic-motivation trait.

These examples indicate that physical education (PE) teachers can use the study’s findings as a teaching strategy: firstly, by drawing on the principles governing the manifestation of these traits during lessons, then they can apply constructive dialogic-interaction methods oriented towards the development of the corresponding trait.

With knowledge of these methods and strategies, PE teachers can successfully apply them within routine lesson activities. In such cases, PE is oriented not only towards pupils’ motor skills but also towards the integrated development of emotional intelligence. This and similar studies confer a dual purpose on PE lessons: fostering the joy of movement and developing emotional intelligence. As noted earlier, the specific nature of a task (e.g., basketball tag, jumping exercises, animal gymnastics) is not the determining factor in developing these traits. Tasks may vary; what matters is that, in performing them, pupils demonstrate the manifestation of intrinsic-motivation and self-confidence traits (as revealed during this study). In such cases, constructive dialogic-interaction methods can be applied.

It is also worth emphasizing that, in a context where ideas of inclusive education are increasingly prominent worldwide, less-experienced PE teachers can shape additional aspects of their practice to include the development of emotional intelligence among pupils with SEN in their lessons, thereby increasing lesson value and quality. Although this study was not conducted in an inclusive education context, its findings are likely to contribute to improving the work quality of early-career specialists and to enhancing pupils’ emotional well-being.

## 5. Conclusions

As our research results suggest various factors may be important for the manifestation of self-confidence: first of all, the teacher’s constructive dialogic interaction, both in terms of emotional support and positive feedback evaluating effort and achievements, and secondly, a subjective sense of success—achievement of an acceptable result; involvement in overcoming challenges.

The manifestation of self-confidence was identified through the observation of a clearly expressed intention to perform the task, active engagement, and positive self-evaluation, including reflective awareness of individual strengths. Students’ understanding of their physical and emotional powers and capabilities can enable them to feel enjoyment and satisfaction; hence, self-confidence likely stimulates intrinsic motivation.

The intrinsic motivation was manifested through active interest, choice of challenging tasks, resolve and perseverance in overcoming challenges, and the need to improve skills and achieve better results.

Constructive dialogic interaction, emphasizing emotional support and strengths-oriented reciprocal teacher–student reflection, is possibly the principal developmental factor for both self-confidence and intrinsic motivation.

## Figures and Tables

**Table 1:** Development factors and indicators of self-confidence and intrinsic motivation traits.

**Contextual Academic Factors**	**Factors that foster self-confidence and intrinsic motivation**	**Themes**			
		**Constructive Dialogic Interaction**	**Manifestation of Self-Confidence**	**Manifestation of Intrinsic Motivation**	**Student’s Intrinsic Self-Motivation Factors**
Educational interactions between teacher and student. Formulating learning goals. Task types. Task instructions. Individualized support. Opportunities to choose alternative (more difficult/easier) tasks. Formulating opportunities Initiation of peer interactions.		***Subthemes** and their indicators*			
		***Emotional support***	***Demonstration of self-efficacy***	***Interest in tasks***	***Self-confidence***
		Encouragement to perform tasks	Clearly expressed desire to perform task	Active engagement	Successful prior experience
		Expression of belief in student efficacy	Active engagement in task performance and awareness of one’s strengths	Choosing challenging tasks	Active engagement
		Highlighting strengths	Positive emotions while performing the task and after success		
		***Arousing self-esteem***	***Positive self-assessment***	***Persistence***	
		Encouraging demonstration of abilities	Reflective awareness of one’s capabilities or strengths and of the result achieved	Effort to perform difficult tasks well	Positive emotions when experiencing success
		Encouraging successful task completion		Determination to overcome difficulties	
		***Positive feedback and evaluation***	Positive evaluation of the effort invested	***Need to improve skills***	***Extrinsic motives***
		Positive appraisal for effort		Demonstrated desire to improve skills	Desire for positive evaluation
		Positive appraisal of results and progress	Positive evaluation of the result achieved	Demonstrated desire to achieve better results	Desire to improve one’s image of demonstrate superiority

## Data Availability

Data available upon request.
